# Cognitive Attachment Model of Voices: Evidence Base and Future Implications

**DOI:** 10.3389/fpsyt.2017.00111

**Published:** 2017-06-30

**Authors:** Katherine Berry, Filippo Varese, Sandra Bucci

**Affiliations:** ^1^Division of Psychology and Mental Health, School of Health Sciences, Manchester Academic Health Science Centre, The University of Manchester, Manchester, United Kingdom

**Keywords:** voice hearing, attachment, dissociation, trauma, psychosis, auditory hallucinations

## Abstract

There is a robust association between hearing voices and exposure to traumatic events. Identifying mediating mechanisms for this relationship is key to theories of voice hearing and the development of therapies for distressing voices. This paper outlines the Cognitive Attachment model of Voices (CAV), a theoretical model to understand the relationship between earlier interpersonal trauma and distressing voice hearing. The model builds on attachment theory and well-established cognitive models of voices and argues that attachment and dissociative processes are key psychological mechanisms that explain how trauma influences voice hearing. Following the presentation of the model, the paper will review the current state of evidence regarding the proposed mechanisms of vulnerability to voice hearing and maintenance of voice-related distress. This review will include evidence from studies supporting associations between dissociation and voices, followed by details of our own research supporting the role of dissociation in mediating the relationship between trauma and voices and evidence supporting the role of adult attachment in influencing beliefs and relationships that voice hearers can develop with voices. The paper concludes by outlining the key questions that future research needs to address to fully test the model and the clinical implications that arise from the work.

## Introduction

Voice hearing (auditory verbal hallucinations) is present in many mental health problems and psychosis in particular (1). Although not necessarily pathological, voices are often associated with distress (2). Over the past three decades, theoretical models have attempted to clarify the underpinnings of these unusual perceptual experiences and inform the development of psychological interventions for distressing voices. Broadly speaking, existing psychological models of voice hearing can be divided into two separate “families” (“vulnerability” models and “distress maintenance” models), depending on the scope and specific aspects of voice-hearing experiences they attempt to examine and explain. Several “vulnerability” models informed by cognitive theory have attempted to identify the psychological/cognitive factors responsible for the formation of hallucinatory experiences. These models include accounts pointing to the importance of a number of putative mechanisms assumed to underpin auditory verbal hallucinations, such as self-monitoring abnormalities (3, 4), source monitoring difficulties (5, 6), and dissociative processes (7). Despite many of these accounts undergoing considerable empirical scrutiny, the “causes” of voice hearing are still largely unknown and there is increasing consensus that a complex interaction between multiple factors rather than single deficits can best account for the vulnerability toward these unusual experiences (8, 9).

In parallel with vulnerability accounts, other psychological models have been proposed to explain why voices are associated with distress and impairment in some individuals but not others [e.g., Ref. (10)]. Arguably the most well-established “distress maintenance” model of voice hearing is the cognitive model of voices, which proposes that the way individuals think about their voice(s) influences their reactions to these experiences (11–14). Consistent with this model, a review of 26 studies found that several types of cognitive appraisals were linked to more distress in voice hearers, including voices appraised as malevolent, powerful, having personal relationship with the individual, and disapproval and rejection toward voices (15).

Vulnerability models are not clear about the factors that differentiate “benign voices” from voices that require intervention. Similarly, psychological distress maintenance models of voice hearing do not offer a suitable explanation of the etiology of these distressing experiences. Another area of inquiry not well delineated by previous models is the extent to which life experiences, and in particular potentially traumatic events (such as experiences of victimization, abuse, and physical and emotional neglect) influence the processes of symptom formation and distress maintenance. The robust association observed between exposure to traumatic life experiences and psychosis more generally (16), and hearing voices more specifically [e.g., Ref. (17, 18)], suggests that trauma represents an important risk factor for voice hearing. Although the specific psychological mechanisms responsible for vulnerability to voice hearing remain unclear, several researchers have proposed, for example, that auditory hallucinations may be conceptualized as trauma-related intrusions [e.g., Ref. (19)] and that several peri- and posttraumatic processes may contribute the formation of hallucinatory experiences, including peritraumatic dissociation and several symptoms of posttraumatic stress [e.g., Ref. (20, 21)].

There is also evidence that trauma exposure may aggravate the psychological processes responsible for voice-related distress and impairment. The cognitive model proposes that beliefs about voices are influenced by the individuals’ life experiences, including trauma and relationships with significant others. For example, Andrew et al. (22) report associations between traumatic life events, including childhood sexual abuse, and negative beliefs about voices (22). Birchwood et al. (23) also present data suggesting that an individual’s perception of being powerless and controlled by others within external social relationships is reflected in the voice/voice-hearer relationship.

In the present article, we outline a recent model of voice hearing informed by both cognitive models of voice hearing and attachment theory (24): the Cognitive Attachment model of Voices [CAV (25)]. The CAV draws on attachment theory to integrate previous vulnerability accounts of voice hearing (trauma-related dissociation and source monitoring accounts) with cognitive and relational models of distress exacerbation and maintenance. In doing so, it aims to (1) explain the complex interplay between contextual and psychological factors that may increase vulnerability to voice-hearing experiences in people who are exposed to adverse life experiences (in particular interpersonal trauma) and (2) understand the psychological processes responsible for variations in how people appraise, respond to, and relate to their voices in ways that may exacerbate and maintain voice-related distress. After presenting a summary of the model, we will elaborate on the theoretical and empirical base regarding the core psychological constructs and processes included in the model and examine the available evidence in support of each hypothesized pathway within the CAV. While we do not present any new data in this paper, we attempt to review existing evidence to test the validity of the model and to highlight opportunities for further research that will help to test the model empirically and progress an evidence-based understanding of the role of putative psychological processes in voice hearing. Finally, we describe clinical implications to illustrate how the CAV model can be used to guide therapeutic work with distressing voices.

## Descriptions of Key Concepts

Prior to presenting the model, we will define key concepts, including attachment, dissociation, and source monitoring, for readers not familiar with the respective literatures.

### Attachment

Bowlby’s (24) attachment theory is one of the most well-established theories of interpersonal relationships. Attachment is an affectional bond, which the individual forms with a significant other, who is approached in response to distress. The theory argues that as a result of their interactions with caregivers during infancy and childhood, individuals develop mental representations of the self in relation to significant others and expectations about how others behave in relationships (26). These “internal working models” guide attention, interpretation, memory, and predictions about future interpersonal interactions. The Strange Situation procedure for assessing attachment behaviors in infancy was crucial in providing empirical support for Bowlby’s theory and measuring individual differences in the quality of attachment relationships to different caregivers. It involves a laboratory-based observation of the infant’s response to two brief separations from, and reunions with his or her caregiver (27).

Although early empirical support for attachment theory came from observations of infants and caregivers, attachment theory is a lifespan developmental theory. In this respect attachment relationships with significant others (most commonly romantic partners) continue to serve an important function in adult lives and attachment working models established in earlier caregiver relationships influence how the individual relates to later attachment figures and regulates negative affect (28). There is evidence of individual differences with respect to adult attachment and some evidence that attachment patterns are stable over time. However, changes in patterns can occur particularly if the individual experiences relationships that are different to their experience of earlier relationships (29).

Unlike attachment in childhood, attachment behaviors in adulthood are most commonly conceptualized in general terms, whereby one has a general attachment style or pattern across relationships, as opposed to an attachment in a relationship with a specific person. However, it is recognized in the literature that people can have different attachment patterns with different people as in childhood (30). Attachment patterns in adulthood are also most commonly assessed using self-report questionnaires or interviews where trained raters ask the person about their experiences of attachment relationships and code attachment patterns based factors such as the coherence of the person’s narrative in describing their experiences (31). In terms of different attachment styles in adulthood, secure attachment is characterized by positive beliefs about the self and others, capacity to regulate affect and form relationships with other people. Conversely, there are insecure attachment patterns, including avoidant and anxious/ambivalent attachment that result from suboptimal experiences of caregiving and are associated with less adaptive interpersonal functioning and affect regulation in adulthood (28). For example, anxious attachment is characterized by negative beliefs about self and sensitivity to rejection from others whereas avoidant attachment is characterized by negative beliefs about others, mistrust of others, and withdrawal from social relationships (28). Individuals who score highly on measures of both anxious and avoidant attachment are conceptualized as having a disorganized pattern of attachment, involving vacillation between approach and avoidance behaviors in relationships, wanting intimacy with others, but fearing rejection and closeness (32).

### Dissociation

The term dissociation refers to a range of clinical and non-clinical psychological phenomena that are relatively common in both the general population as well as individuals with clinically significant mental health difficulties [e.g., Ref. (33)]. Often defined as the “lack of normal integration of thoughts, feelings and experiences into the stream of consciousness and memory” [(34), p. 727], dissociation represents the core component of several DSM-5 several (35), most notably dissociative disorders (e.g., dissociative identity disorder, dissociative amnesia, and depersonalization/derealization disorder), but also specific trauma and stress-related presentations (in particular the dissociative subtype of posttraumatic stress disorder (PTSD) introduced in the latest revision of the DSM). A widely accepted unitary conceptualization of dissociation assumes that different dissociative experiences lie on a single continuum of severity ranging from the relatively benign forms of absorption and other fleeting dissociative states frequently experienced in non-clinical populations, to more pervasive experiences of depersonalization, derealization, and identity alteration that can sometimes cause severe distress or discomfort. More recently, several authors (36, 37) have proposed that the different experiences traditionally described with the term dissociation may reflect two qualitatively distinct classes of phenomena, namely, detachment (which encompasses derealization, depersonalization, and similar experiences characterized by a sense of separation or detachment from aspects of everyday experience) and compartmentalization phenomena (such as dissociative amnesia and other symptoms that allegedly result from reversible disruptions in normal processes for the monitoring and control of mental experiences, resulting in the functional or perceived “separation” of certain elements of one’s current experience and mental functioning). Due to the current lack of convincing research clarifying whether voice hearing is more robustly related to specific dissociative experiences, in the context of the current paper, we use the term dissociation broadly to describe the altered states of consciousness captured by multifactorial measures that are used widely in both research and clinical settings [e.g., the Dissociative Experiences Scale (DES)].

Although it can be experienced in the absence of a history of trauma, dissociation is frequently observed in the immediate aftermath of traumatic events, and trauma survivors can sometimes experience a heightened predisposition to dissociative many years after the original traumatic event [e.g., Ref. (38)]. The alleged link between trauma and dissociation has been the subject of extensive theoretical debate and empirical scrutiny, with some regarding this association as spurious or artifactual, and others as consistent with the alleged action of an in-built “defense mechanisms” that allows people to reduce the overwhelming emotional and cognitive consequences of traumatic experiences [for a critical appraisal of these theoretical debates and associated empirical research, see Ref. (38)].

### Source Monitoring

The cognitive processes that might ultimately account for the genesis of hearing voices and other hallucinatory experiences are still unclear. However, there is some agreement that they are the result of the misattribution of internally generated cognitive events (e.g., inner speech) to sources that are alien or external to the self [e.g., Ref. (5, 39–41)]. Several cognitive accounts have assumed that specific anomalous cognitive processes may underlie this misattribution. Bentall (5) proposed that the origin of hallucinatory experiences can be explained in terms of source monitoring (also described in some papers with the term “reality discrimination”), a metacognitive process used to discriminate between internal and external perceptions and make attributions about the origin of mental experiences. This account argues that hallucination-prone individuals are less able to discriminate between internally and externally generated cognitive events and have a bias toward misattributing internal cognitive events to external sources. A wealth of studies using various experimental procedures, including signal detection [e.g., Ref. (42)], self-monitoring [e.g., Ref. (43)], and source memory [e.g., Ref. (44)] paradigms, has provided strong empirical support for the proposal that source monitoring biases might underpin a vulnerability toward hallucinatory experiences. In a meta-analysis of this literature, Brookwell et al. (6) found that these source monitoring biases are evident both clinical and non-clinical hallucination-prone individuals (i.e., hallucinating patients or non-clinical participants with high scores on hallucination-proneness measures) when compared to non-prone sample samples. The exact etiology of such biases, however, and the extent to which they might be influenced by environmental risk factors for hallucinations and other psychotic experiences (e.g., trauma exposure), remains to be clarified.

## The CAV

**Figure 1:**
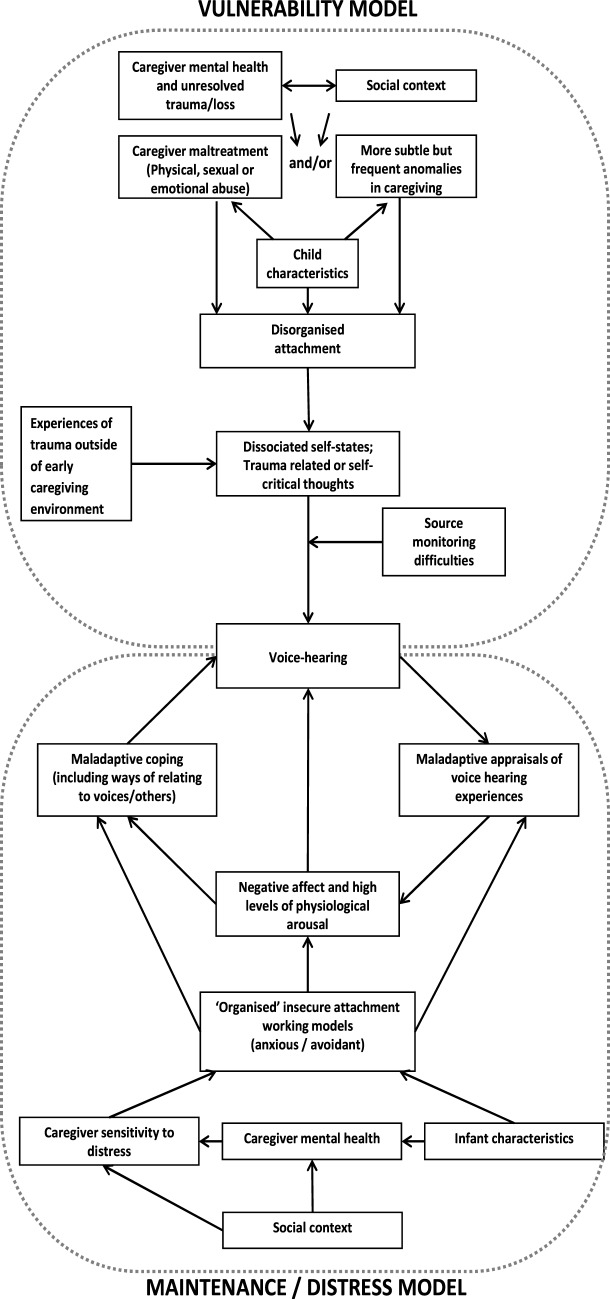
Graphical representation of the cognitive attachment voice-hearing model [adapted from Ref. (25)].

The CAV model draws on cognitive, attachment, and dissociative processes to explain the development and maintenance of distressing voice hearing (Figure 1). Further to the original publication of the CAV model (25), in this paper, we more clearly delineate the vulnerability and distress maintenance aspects of the CAV model, which we will now describe in more detail. Essentially, in line with cognitive and relational models of psychosis, the CAV proposes that disorganized attachment, coupled with dissociative and cognitive processes, can help explain the link between trauma and voice hearing. Insecure internal working models, combined with appraisals, influence affective emotional and behavioral reactions to voice-hearing experiences. As a caveat, readers should note that the CAV proposes one route to the development of voice hearing and the maintenance of voice-related distress; we are not purporting to explain all voice experiences.

### Vulnerability Components of the CAV Model

In line with previous accounts of voice hearing, the CAV proposes that voices can be understood as dissociated components of the self or “compartmentalized” trauma-related intrusive memories [e.g., Ref. (7, 19, 45)] (see top circle, Figure 1). In some predisposed individuals, these internal states and cognitive events are experienced and/or interpreted as external and current rather than internal and memory based, possibly as a result of biased source monitoring processes that have been shown to underpin hallucination proneness in numerous clinical and non-clinical studies [e.g., Ref. (6, 46)]. A central tenet of the CAV model is that the propensity to experience dissociative states is driven or exacerbated by a specific type of attachment pattern, namely, disorganized attachment. This attachment pattern was originally observed in the context of infant research where some infants were found to display disorganized and disoriented responses to separation and reunion from caregivers that appeared contradictory and inconsistent with the “organized” attachment patterns such as avoidant or anxious/ambivalent attachment previously identified [e.g., Ref. (47)]. Disorganization is the outcome of interactions in which the infant experiences the attachment figure as frightening, frightened, or dissociated in times of stress. The caregiver might act in ways that are confusing and unpredictable for the infant, rendering it difficult for them to develop an “organized” pattern of self-protection. According to Liotti (48), the infant experiences “fright without solution” at being confronted with the biological paradox that the attachment figure, the primary source of safety and protection, is also the source of the infant distress. There is evidence of associations between attachment disorganization and parental maltreatment (49), but the development of a disorganized attachment pattern could also be influenced by more subtle (but frequent or pervasive) disruptions in parental attunement, possibly caused by a range of adverse conditions and circumstances [e.g., parental poor mental health, trauma, and experiences of loss (49–54)]. Critically, disorganized attachment has been identified as a developmental antecedent of dissociation in response to later trauma (48, 55–57), a factor believed to contribute to one’s predisposition to experience hallucinations [e.g., Ref. (46, 58)].

### Distress and Maintenance Component of the Model

In addition to implicating disorganized attachment in the processes leading to the formation of hallucinatory experiences, the CAV model proposes a distress maintenance cycle, whereby “organized” but insecure attachment working models influence the appraisals and cognitive-behavioral responses that could exacerbate voice-related distress and contribute to the maintenance of distressing voice-hearing experiences (see bottom circle, Figure 1). In terms of the distress maintenance cycle of distressing voice hearing, we suggest that, once voices develop, insecure attachment styles (which are influenced by early relational caregiver experiences) could also influence how voices are appraised, the way in which different voice hearers relate to their voices, and the subsequent cognitive-behavioral strategies that different voice hearers employ to control these experiences. While for the purpose of the model it appears that attachment patterns are categorically assigned to individuals, we recognize that there can be considerable overlap in attachment patterns and that allocating people into a specific attachment “category” is somewhat artificial; individuals can display characteristics associated with various attachment patterns (59).

Drawing from attachment and cognitive theories, the CAV predicts that high levels of anxious attachment in voice hearers’ relationship with their voices, or a general anxious attachment style (i.e., an attachment style characterized by beliefs that they need to rely on other people, negative beliefs about the self and an expectation that other people will let them down), will result in beliefs that voices are powerful, but also fluctuating beliefs about voice benevolence and malevolence. The voice-hearer relationship is likely to be characterized by hearer dependence and voice dominance, and the individual is likely to be hypervigilant and sensitive to the voices reaction to them. For example, in the context of command hallucinations, the hearer may follow through with the voice(s) command to please or appease the voice, but at the point that commands become more unreasonable or in conflict to a high degree with the hearer’s core values, s/he may fear rejection and even punishment. Those with anxious attachment styles and access to alternate attachment figures in the external social world will also increase proximity to, and dependence on, these individuals in response to voice hearing-related distress. Furthermore, an anxious attachment style would be associated with negative beliefs about the individual’s capacity to cope with the voice. In contrast, individuals with high levels of avoidant attachment in their relationships with their voices or relationships more broadly may hold malevolent beliefs about voices and may suppress and/or resist the voice, with this avoidant response ultimately maintaining negative beliefs and voices over time. For example, a hearer with avoidant attachment may believe that voices cannot be trusted and either attempt to distract themselves from the experience or fight back by being hostile and aggressive in response. A general avoidant attachment may serve to maintain voice-related distress by reducing the probability that individuals will develop new attachments or utilize social supports to help cope with distress.

In summary, attachment style influences the maintenance of voices *via* two routes: an engagement route (anxiously attached voice hearers may tend to “actively seek” the voices) and a suppression route (people employing different suppression strategies that ultimately fail *via* rebound effects). Within the model, disorganized attachment is conceptualized as a *precursor* to voice hearing. However, as the concept of disorganized attachment has been conceptualized in terms of high levels of anxious and avoidant attachment, it could be hypothesized that some individuals oscillate between an anxious and avoidant pattern of relating to voices.

## Evidence to Support the Model

### Early Relational Trauma and Attachment

Attachment theory argues that insecure attachment styles arise from adverse childhood experience. Suboptimal caregiving, including both subtle but frequent disruptions in caregiving and more extreme experiences of neglect and abuse, has all been identified as predictors of attachment difficulties (60, 61). In terms of associations between specific types of experience and different attachment patterns, anxious attachment (or ambivalent attachment in infancy) has been associated with inconsistent parenting, typified by over intrusiveness and overt expressions of devotion followed by neglect (62). As the attachment figure’s responses are inconsistent, as opposed to completely rejecting, the preoccupied individual is hypothesized to develop a strategy of increasing negative affect in an attempt to elicit attention (28). Avoidant attachment has been associated with more consistent levels of neglect or uncaring and critical behavior from caregivers (63). As indicated above, there is evidence of associations between attachment disorganization and parental maltreatment (49), but the development of a disorganized attachment pattern could also be influenced by more subtle (but frequent or pervasive) disruptions in parental attunement, possibly caused by a range of adverse conditions and circumstances [e.g., parental poor mental health, trauma, and experiences of loss (49–54)].

These relationships have not been tested through longitudinal research in voice hearers, but a number of cross-sectional studies in psychosis report associations between earlier caregiving and insecure attachment (64–66). For example, in a sample of 80 people, Berry et al. (64) found a negative relationship association between participant reports of neglectful parental care and insecure avoidant attachment. The authors also compared levels of attachment across four different groups: 26 participants reporting trauma with significant others in childhood; 12 participants reporting trauma with significant others in adulthood only; 28 participants reporting traumatic interpersonal events involving non-significant others; and 14 participants reporting no traumatic interpersonal events. The groups differed in terms of attachment anxiety and there were higher levels of anxious attachment in people who experienced trauma with significant others in childhood. Unexpectedly, there were no group differences for attachment avoidance. Avoidant attachment has been characterized by a tendency to underreport distress (67), thus making it difficult to detect possible associations between interpersonal trauma and avoidant attachment. As discussed below, these associations between trauma and anxious attachment have been replicated in a recent study with voice hearers that also attempted to test a mediation model whereby insecure attachment mediates associations between earlier trauma and dimensions of voice hearing (68). Finally, in the largest study examining attachment profiles in psychosis to date, data from 588 participants who met criteria for non-affective psychosis showed that a disorganized attachment pattern was associated with a higher proportion of sexual and physical abuse and more severe positive symptoms compared to other attachment patterns (69), suggesting that disorganized attachment is a more putative attachment style compared to other types of attachment for positive symptoms. However, despite relatively robust evidence of associations between earlier relational trauma and attachment, it is important to note that not all people who have experienced trauma develop problematic attachment patterns [e.g., Ref. (54)], suggesting that other biological, psychological, or social resilience factors may be important in predicting later outcomes including the development of psychopathology and distressing voices.

### Trauma and Dissociation

Despite the popularity of proposals arguing that the apparent link between dissociation and adverse life experiences may be artifactual, and accounted for by the high levels of fantasy proneness and other cognitive distortions observed in individuals with dissociative experiences [e.g., Ref. (70)], there is considerable evidence suggesting that dissociation is a pervasive psychological sequela of traumatic life experiences (38). For example, the findings of cross-sectional studies suggesting a robust association between histories of childhood abuse and adult dissociation [e.g., Ref. (71)] have been corroborated by a growing number of prospective investigations and studies that sought objective confirmation of self-reported trauma histories [for a review, see Ref. (38)]. Although heightened dissociation has been linked to a wide range of traumatic life experiences, empirical studies indicate that the risk of experiencing pervasive dissociative symptoms is particularly elevated in individuals exposed to severe interpersonal traumas, such as acts of abuse, maltreatment, or victimization perpetrated by close family members or significant others. For example, the findings of studies conducted in the context of Betrayal Trauma Theory, a theoretical model that views dissociation as an adaptive response that minimizes the distress and conflict faced when perpetrators are also the very same persons and/or institutions on which the victim depends for his/her survival or well-being (72), suggests that associations between dissociative phenomena and interpersonal traumatic experiences characterized by high interpersonal closeness is particularly robust relatively to those between dissociation and other types of traumas [e.g., Ref. (73, 74)]. In the light of this evidence, traumatic events that occur in the context of salient attachment relationships can be regarded as particularly influential to the development of dissociation and related phenomena, including hallucinatory experiences, as explained in subsequent sections.

Recent meta-analytic studies indicate that dissociative experiences are common among people experiencing psychosis (75), a finding that is not surprising in the light of the plethora of evidence reflecting high prevalence of interpersonal traumatic life experiences in this clinical population [e.g., Ref. (46)]. Several empirical studies suggest that the heightened levels of dissociation reported by people with psychosis relative to non-psychiatric controls are explained by trauma exposure [e.g., Ref. (46, 76, 77)], and therefore are unlikely to simply be a byproduct of psychotic symptoms presence. Similar findings were obtained in studies that specifically considered samples of clinical and non-clinical voice hearers [e.g., Ref. (46, 78–80)], with some notable non-replications (81, 82) that are possibly explained by a number of methodological drawbacks in these studies [insufficient statistical power and/or variance in trauma exposure; use of crude measures of trauma exposure; see Ref. (58)].

### Attachment and Dissociation

Liotti (83) suggested that if infants experience interactions with parents exhibiting frightened/frightening behaviors as a result of their own unresolved loss or trauma this can create long-term vulnerabilities to dissociative disorders. Liotti (48) proposes that disorganized attachment in infancy reflects incoherent and confusing emotional and behavioral coping strategies in which the person is unable to resolve the conflict between simultaneously seeking safety from their attachment figure and avoiding distress from them. Liotti (48) suggests that in adulthood, when faced with a stressor, these incoherent coping strategies are activated causing reactions that mirror dissociative experiences in which an individual is unable to coherently integrate memories, consciousness, and self-identity. Although a detailed discussion of the neurobiology of attachment and the neurological effects of trauma is beyond the scope of this paper, it is possible that repeated exposure to relational traumas in childhood results in oversensitivity to threat in the context of later stressors, as smaller hippocampal and frontal volume lead to a predominance of amygdala-driven processing rather than hippocampal or frontal cortex-driven processes (84).

There is evidence of associations between reports of parental loss and later dissociation/absorption in offspring (83, 85). Prospective, longitudinal studies have found similar results in terms of developmental pathways to dissociation [e.g., Ref. (56, 57, 86, 87)]. For example, Ogawa et al. (57) examined dissociative behaviors and their relation to self-organization in 168 young adults at high risk of poor developmental outcomes due to poverty using a longitudinal design, which assessed traumatic life events, attachment quality, adaptational functioning, and dissociative symptomatology at five time points from birth to 19 years. In addition to age of onset, severity, and chronicity of trauma, disorganized and avoidant attachment to parents was a significant predictor for scores on all the measures of dissociative phenomena. Surprisingly, few studies have investigated associations between adult attachment and dissociation. One study investigating PTSD and dissociation in victimized female college students with and without a history of childhood abuse found that self-reported secure attachment style was negatively related to dissociation as measured by the DES [the most widely used questionnaire measure of dissociative experiences (34)], and self-reported preoccupied and fearful attachment were positively associated with dissociation (88). When victimization/abuse and the four attachment scores were entered into the same model, only fearful attachment uniquely contribution to dissociation. Dismissing-avoidant attachment was not significantly related to dissociation. The authors propose that their findings are consistent with Liotti’s (83) view that dissociation is better understood as a form of fear-based disorganization rather than emotional detachment. Although the study did not directly measure disorganized attachment, it has previously been argued that fearful attachment as assessed on self-report adult attachment questions has conceptual overlap with the concept of disorganized attachment in infancy and unresolved attachment on the Adult Attachment Interview (AAI) (32).

### Dissociation, Source Monitoring, and Voices

There have been several proposals arguing that dissociation could represent a candidate process to explain the well-replicated association between trauma and the predisposition to experience psychotic symptoms, in particular hearing voices [e.g., Ref. (45, 89, 90)]. In recent years, a growing body of cross-sectional studies has examined the association between dissociative states and voice-hearing experiences across several clinical and non-clinical populations. Our recent systematic review and meta-analysis of this literature found robust associations between voice hearing and dissociation, which were observed not only in research with individuals with a diagnosis of schizophrenia and related psychoses but also in PTSD, DID, and non-clinical studies (58). There is also growing empirical evidence suggesting that dissociation mediates the relationship between childhood trauma and the proneness to hearing voices, a finding that has been already been independently replicated in both clinical (46, 79, 91) and non-clinical samples [e.g., Ref. (91, 92)]. In a recent prospective study, Geddes et al. (93) found that peritraumatic dissociation (meaning, the extent to which the victim dissociated during the traumatic event), in conjunction with other peritraumatic and trauma-related psychological variables, predicted the onset of hallucinatory experiences in survivors of interpersonal assaults. The exact processes through which dissociation might lead to the formation of hallucinatory experiences, and the extent to which the cognitive underpinnings of dissociation interact or overlap with those that promote hallucinatory experiences, remain a matter of theoretical debate as very few empirical investigations have attempted to tackle these research questions. In a rare study of this kind, Varese et al. (46) tested service users with psychosis on self-report measures of dissociation and experimental measures of reality discrimination (source monitoring). In this study, hallucinating individuals reported significantly higher levels of dissociation compared to both service users who were currently hallucination-free but had suffered from hallucinations in the past (in other word, individuals whose hallucinations were in remission), and service users with diagnoses in the schizophrenia spectrum who had *never* experienced hallucinations at any point of their lives. In contrast, the analyses of reality discrimination data indicated that the performance of hallucinating individuals and remitted hallucinators was significantly (and equally) more biased relative to service users who never experienced hallucinated perceptions. These findings suggest that heightened dissociation only characterized participants who were currently suffering from hallucinations rather than representing an enduring trait, whereas reality discrimination difficulties could be regarded as a trait-like vulnerability present in both current and remitted hallucinating individuals. Additional analyses of these data indicated that dissociation was unlikely to be directly responsible for the reality discrimination difficulties assessed in the context of this study; the reality discrimination scores of service users who presented with “pathological” levels of dissociative symptoms (94) were in fact no different than those of “non-dissociative” service users. Varese et al. (46) argue that this may reflect a possible “two-hit” model, in which dissociation triggers voices in those vulnerable to reality discrimination (or source monitoring) difficulties. In the lack of better evidence, the current representation of the CAV model reflects and incorporates the findings of this study, but we recognize that further investigations in this area are required to corroborate or clarify the interplay between dissociation and the cognitive underpinnings of hallucinatory experiences.

### Attachment and Voices

There is substantial evidence that insecure attachment increases vulnerability to the development of mental health problems (28). For example, there is evidence from prospective research that insecure attachment increases vulnerability to PTSD (95) and mediates associations between childhood maltreatment and later anxiety and depression (96).

More specifically in the context of psychosis, Ponizovsky et al. (97) and Berry et al. (98) investigated adult attachment and symptoms in participants diagnosed with schizophrenia or related psychoses. Both research groups found associations between attachment anxiety and voices, but Ponizovsky and colleagues (97) also found relationships between attachment avoidance and voices. Bentall et al. (99) explored the issue of symptom specificity by examining relationships between childhood adversities and both paranoia and hallucinations with data from Adult Psychiatric Morbidity Survey. Using analyses that controlled for the covariation between paranoia and hallucinations, the authors found that childhood rape was specifically associated with hallucinations whereas being brought up in institutional care was associated with paranoia, but not with hallucinations. The authors argue that severe trauma increases vulnerability to voice hearing, whereas attachment disturbance increases susceptibility to paranoia. Wickham et al. (100) explored associations between attachment, paranoia, and hallucinations in people diagnosed with a schizophrenia-spectrum disorder and non-clinical controls. Similar to the findings of Bentall et al. (99), attachment was not correlated with hallucinations when controlling for paranoia.

However, research to date has focused on insecure attachment patterns and their association with psychotic symptoms as a disorganized attachment pattern has been difficult to measure using self-report measurement tools. However, researchers have long made conceptual links between disorganized attachment and voice-related distress, suggesting that high scores on the two organized attachment dimensions likely reflect a disorganized attachment pattern. Yet, specific correlates of disorganized attachment more specifically have only very recently been investigated. To the best of our knowledge, there are two studies that have attempted to explore disorganized attachment and voice-hearing correlates.

Using a covariance modeling approach to explore associations between attachment and non-clinical psychotic phenomena, MacBeth et al. (101) found that paranoia was predicted by an “organized” model consisting of attachment anxiety, avoidance, and interpersonal distancing strategies, whereas hallucinations were predicted by a more complex model represented by a combination of attachment anxiety and interpersonal affiliating strategies with attachment avoidance and interpersonal distancing strategies. The authors argue that the hallucinations model characterizes the contradictory and competing interpersonal strategies of a disorganized attachment pattern.

Bucci and colleagues (69) combined observations of 588 psychosis patients on the Psychosis Attachment Measure (98), the most frequently used self-report attachment measure in psychosis research, to examine associations between attachment patterns and positive psychotic symptoms (hallucinations and delusions). In line with CAV predictions, Bucci et al. (69) found that those with more frequent reports of trauma history, in particular sexual and physical abuse, and more frequent positive psychotic symptoms, were assigned to a disorganized attachment class using latent profile analysis.

### Attachment, Beliefs about/Ways of Relating to Voices, and Voice-Related Distress

Berry et al. (102) explored associations between attachment and the nature of the person’s relationship with voices. The authors found evidence of associations between avoidance in attachment relationships and themes of rejection, criticism, and threat in relationships with voices. This study was novel in investigating attachment and voice hearing but was limited by the fact that themes from symptom assessments were used to derive the content of voices; participants were not specifically asked about the nature of their relationships with voices.

In a sample of 44 voice hearers, Robson and Mason (103) found that avoidant attachment was associated with voice intrusiveness (voice perceived by hearer as intrusive) and hearer distance (hearer relates to their voice from a position of distance), and anxious attachment was related to voice intrusiveness and hearer dependence (hearer relates to their voice from a dependent position). Furthermore, the relationship between insecure attachment and voice-related distress was mediated by voice malevolence and omnipotence. In a similar study involving 55 voice hearers, Pilton et al. (68) found that anxious attachment was associated with voice-related severity and distress and further dimensions of voice hearing including: voice omnipotence, voice dominance, voice intrusiveness, and hearer dependence, but there were no associations between avoidant attachment and voices. Pilton et al.’s (68) mediation analyses showed that anxious attachment mediated the relationship between childhood trauma and voice dimensions including voice-related severity, voice distress, voice malevolence, voice omnipotence, voice resistance, and hearer dependence. Consistent with Robson and Mason’s (103) research, further mediation analyses also found that the relationship between anxious attachment and voice-related distress was mediated by beliefs about voices including voice malevolence, voice omnipotence, and voice resistance. These findings suggest that anxious attachment may have a direct effect on voice-related distress but also influence voice-related distress *via* negative beliefs about voices and maladaptive ways of responding to voices.

The idea that people form specific attachments with voices also raises the question whether or not relationships with voices can be conceptualized as attachment relationships. There is evidence that, for some people, voices do provide a source of comfort from distress as well as companionship and that people anticipate and experience a sense of loss following reductions in voice hearing and when their voices are not present (104, 105). Speculatively, it may be particularly likely that voices take on an attachment quality if other social relationships diminish following the onset of psychosis.

## The Added Value of the Model

We recognize that there may be multiple routes to voices, but our model aims to clarify the nature of one of these possible pathways. The unique aspect of the CAV is the concept of attachment, and specifically the role of disorganized attachment and dissociative processes, in helping to explain the association between trauma and voice hearing. Although previous theories have highlighted the importance of trauma-related dissociation in voice hearing, one of the strengths of attachment theory is that it not only highlights the role of overtly abusive events but also emphasizes the role of more subtle childhood experiences in influencing the way individuals manage distressing emotions and relate to other people (106), which in turn may also influence susceptibility to voices.

A further advantage of the CAV is that it integrates vulnerability and maintenance distress models of voice hearing. Within the model disorganized attachment patterns increase vulnerability to hear voices, but secondary attachment strategies including insecure avoidant and anxious styles attachment styles influence belief appraisal processes, affect emotional regulation strategies and interpersonal relationships with both others in the social world and voices, once voices develop. Relatedly and perhaps less well articulated in the literature is the notion of secure attachment. Our own data suggest that a substantial proportion of people with psychosis have secure attachment styles (69), and our model predicts that such individuals would develop more psychologically healthy ways of thinking about and relating to their voices and seeking support from others in their social world, including mental health services. The CAV therefore has the potential to predict resilience to distressing voices as well as vulnerability and maintenance.

## Limitations and Further Research

Further research is needed to investigate associations between specific childhood adversities and attachment, including disorganized attachment, which is not well captured by self-report measures of attachment styles. Bucci et al. (69) describe using a simple self-report measure of attachment to derive a measure of disorganized attachment using latent class analysis, but arguably we need processes for measuring disorganized attachment that more directly assess the concept and are more feasible to use in the context of large research studies than the AAI (107).

A more nuanced understanding of dissociation is also needed in the psychosis literature. Current definitions of dissociation are rather global and general whereby dissociation has traditionally been conceptualized as a unitary phenomenon (37). For example, the broad use of the term dissociation is often used to describe experiences ranging from relatively benign forms of psychological disengagement (e.g., absorption) to more pervasive dissociative experiences such as derealization, depersonalization and identity alteration. However, the concept of a dissociative continuum has been criticized for being overly generic (37). For example, Holmes et al. (36) argue that there are two different kinds of dissociation: detachment and compartmentalization. Detachment is defined as a state of consciousness that is removed from everyday experience and caused by a neurobiological response to threat (108). Compartmentalization is defined as an inability to deliberately control cognitive processes or behaviors that would normally be controllable and is caused by disturbances in processes underlying consciousness and mental control (109, 110). It is as yet unclear if disorganized attachment predisposes individuals to develop specific dissociative phenomena. Similarly, it is unclear which aspects of dissociation are important in determining the development of voice hearing. Whereas existing measures, such as the DES (34), might be regarded as well suited for the assessment of prototypical examples of dissociative detachment (e.g., experiences of depersonalization and derealization), no comprehensive measure of dissociative compartmentalization has been developed yet. This line of research will require the development of novel, purposely designed and psychometrically robust measures to assess dissociative detachment and compartmentalization. Furthermore, existing research has predominantly conceptualized AVHs as a unitary construct, despite indications that they may be phenomenologically heterogeneous experiences. Like in the case of dissociative experiences, researchers have proposed the existence of phenomenologically distinct subtypes of hearing voices, which are possibly characterized by different psychological underpinnings and might require different treatment approaches [e.g., Ref. (111–115)]. Research carried out to identify these phenomenologically distinct subtypes is, however, in its infancy. As this line of research develops, it will be possible to clarify whether the psychological processes outlined in the CAV model are applicable across different types of voices, or may be particularly implicated in certain subtypes (e.g., dialogical voices characterized by a specific identity, as voice hearers may be particularly prone to respond to these voices according to their attachment styles).

Finally, there is a growing call to examine resilience factors within the context of trauma and voice hearing. Secure attachment, which is shaped by biological, psychological, and social influences, may be an important resilience factor that both influences the likelihood of experiencing dissociation in response to trauma and/or how adaptively people respond to the voice hearing once it develops. Indeed, evidence that secure attachment is a potentially important resilience factor in preventing the development of PTSD following exposure to trauma (95), suggests it would be important to examine if these findings generalize to other trauma-related symptoms, such as dissociation and psychotic symptoms.

## Clinical Implications

The model presented here has a number of clinical implications. First, the proposed model highlights the importance of asking voice hearers about their histories of relational trauma and experiences in attachment relationships (116, 117). Staff are likely to need further training and supervision in how to sensitively ask about, and response to, distressing disclosures of abuse and neglect in a psychologically informed way (117, 118). The CAV also emphasizes the overlap between relationships with voices and broader social relationships. This has the advantage of normalizing voice hearing and suggests that therapeutic strategies that have been shown to improve relationships in the social world might also be beneficial in voice hearing (119). In addition, Bowlby (120) conceptualized the therapeutic relationship as an attachment relationship and argued that effective psychotherapeutic intervention can provide an alternative interpersonal experience termed “corrective emotional experience,” affording individuals the opportunity to develop a broader range of interpersonal behaviors. Therapist sensitivity, responsiveness, reliability, and consistency are key to the development of a secure therapeutic base. In the context of voice hearing, once a “secure base” has been established and the person is willing, individuals may feel more comfortable to explore and process the links between previous relationships and relationships with voices. Perhaps most fundamentally, the possible role of attachment in voice hearing highlights the likely benefits of interventions that help children foster secure attachments and therefore stronger resilience to voice hearing in the first place. Indeed, randomized control trials demonstrate the effectiveness of attachment-based interventions for maltreating families (121). If disorganized attachment is critical in the developmental pathway of voice hearing, early identification of this vulnerability is crucial as working with a disorganized attachment patterns takes time, consistency, and persistence. Reliable and consistent boundaries are essential; consistent and predictable staff members and service response to incidents over time are needed to make it possible to develop a corrective attachment experience for individuals (122).

Given the finding that a significant proportion of voice hearers do have secure attachment styles [e.g., Ref. (69)], identifying resilience factors and adopting a strengths-based approach may also be important in clinical work and intervention development.

While it could be argued that some of the above implications are also indicated by the existing cognitive model of voice hearing, such as the importance of asking about trauma and exploring how the meaning from these experiences influences relating to voices, in line with the previous section, we argue that an attachment perspective provides added value both clinically and theoretically. Most notably, the fact that attachment theory is a universal theory of relationships, which applies to both voice hearers and mental health workers alike and emphasizes the functional nature of insecure attachment styles in the context of earlier relationships means that it has the potential to be less pathologizing than other models. Relatedly, attachment theory also provides a framework for conceptualizing the role of the mental health workers’ own relational histories and attachment styles within the therapeutic processes, including how these interact with those experiences and attachment patterns of voice hearers (122, 123).

## Conclusion

It is important to understand the psychological mechanisms underlying voices so this can inform the development of psychological therapies for distressing voices (124). Here, we argue that the CAV model identifies the way in which the psychological mechanisms of attachment and dissociation might develop cognitive theories of voices. Other researchers have developed theories about the role earlier experiences in voice hearing without drawing on attachment theory, and other models have noted the importance of voices being understood as an interpersonal relationship, but this is the first attempt at integrating attachment theory within cognitive and dissociative frameworks for understanding voices.

Attachment theory does not, in itself, explain all instances of voice hearing- or voice-related distress. Nevertheless, we hope that we have argued that attachment theory, a key theory of emotional regulation and interpersonal relationships, should not be ignored when developing an understanding of how individuals cope with voice-related distress and relate to the voice-hearing experience.

## Author Contributions

All authors—development of ideas and writing paper.

## Conflict of Interest Statement

The authors declare that the research was conducted in the absence of any commercial or financial relationships that could be construed as a potential conflict of interest.
